# Static Bilateral Plantar Load Sharing One Month After Total Knee Arthroplasty for Varus Osteoarthritis: A Prospective Pedobarographic Equivalence Study

**DOI:** 10.3390/medicina62101840

**Published:** 2026-09-23

**Authors:** Oğuz Şimşek, Recep Altın, Bilge Kağan Yılmaz, Emre Tosun, Mevlüt Özbahçe

**Affiliations:** 1 Department of Orthopedics and Traumatology, Faculty of Medicine, Afyonkarahisar Health Sciences University, 03030 Afyonkarahisar, Türkiye; 2 Department of Orthopedics and Traumatology, Denizli Servergazi State Hospital, 20010 Denizli, Türkiye

**Keywords:** total knee arthroplasty, mechanical alignment, plantar load distribution, pedobarography, knee osteoarthritis, static stance, cartilage mechanobiology, equivalence testing

## Abstract

*Background and Objectives*: Total knee arthroplasty (TKA) reduces coronal malalignment, but whether this correction redistributes load at the foot early after surgery is unknown. We evaluated static plantar load distribution before and one month after TKA and tested whether the magnitude of axial correction predicted the magnitude of load change. *Materials and Methods:* Sixty-four consecutive patients undergoing unilateral TKA for advanced knee osteoarthritis were enrolled prospectively; 60 completed both assessments. Hip–knee–ankle (HKA) angle, Q angle, and passive extension deficit were recorded preoperatively and at a mean of 30.9 ± 3.3 days postoperatively, together with static pedobarography of both feet in a bilateral stance under an instruction to load both limbs symmetrically. The primary outcome was the relative bilateral load share of the operative foot. Change scores were analyzed with paired tests, two one-sided equivalence tests (TOSTs), correlation, multivariable regression, and a linear mixed model. *Results:* The HKA angle improved from 10.79 ± 4.24° to 3.31 ± 3.51° of varus (*p* < 0.001), although residual varus persisted in 47 of 60 patients. Load share of the operative foot did not change significantly (−1.74 percentage points, 95% CI −4.92 to +1.45; *p* = 0.247), and equivalence was demonstrated within the primary ±5-percentage point margin (TOST *p* = 0.022) but not within an exploratory ±3-point margin (*p* = 0.216). In a sensitivity analysis excluding seven patients with comorbidities or previous osteotomy (*n* = 53), equivalence within ±5 points was not demonstrated (*p* = 0.094). Regional load share and mean pressure were likewise unchanged. No statistically significant relationship was demonstrated between correction magnitude and the change in load share (r = 0.222, *p* = 0.088; adjusted β = 0.428 percentage points per degree, 95% CI −0.598 to 1.455; *p* = 0.407), although the confidence interval was wide. *Conclusions:* One month after TKA for advanced, predominantly varus osteoarthritis, static bilateral plantar load sharing measured under an instruction to load both limbs symmetrically was equivalent to its preoperative state within ±5 percentage points. This equivalence may in part reflect the standardized instructions rather than the absence of a biological effect. No relationship between the degree of coronal correction and load redistribution was demonstrated. These findings are confined to the first postoperative month and to an instructed symmetrical static stance. Dynamic loading, gait, and later adaptation were not assessed.

## 1. Introduction

Knee osteoarthritis is among the most common degenerative joint diseases worldwide and a major cause of pain, functional limitation, and reduced quality of life [1,2]. When conservative management fails, total knee arthroplasty (TKA) is an effective procedure for pain relief, deformity correction, and restoration of mobility [3].

Osteoarthritis is increasingly understood as a disease in which mechanical and biological factors are inseparable. Articular cartilage depends on physiological cyclic loading to maintain the proteoglycan-rich matrix and collagen network that give it its viscoelastic, load-distributing properties [4]. Chondrocytes sense their mechanical environment through integrins, the primary cilium, and mechanosensitive ion channels such as PIEZO1 and TRPV4. Loading within the physiological range is anabolic and anti-inflammatory, whereas supraphysiological or abnormally distributed stress shifts chondrocyte signaling towards catabolic and pro-inflammatory pathways, with loss of proteoglycan, disruption of the collagen network, and progressive deterioration of the mechanical properties of the tissue [4,5]. This shift is further modulated by systemic and local biochemical factors, including inflammatory mediators and micronutrient status [5,6]. Coronal malalignment is the clearest clinical expression of abnormal load distribution. Varus deviation concentrates the load line on the medial compartment, and the resulting mechanical overload accelerates the degenerative cycle [1,7]. If TKA is intended to interrupt this cycle by restoring a physiological load path, whether and when the corrected axis is expressed in the load transmitted through the foot is a question of direct mechanobiological relevance.

A principal objective of TKA is restoration of lower extremity alignment [8]. The knee, however, is not an isolated structure but a central element of the lower extremity kinetic chain, interacting with the hip, ankle, and foot during standing and gait [9,10]. Alteration of knee alignment may therefore influence load transmission at more distal segments.

Varus malalignment is the most frequent deformity in advanced knee osteoarthritis and increases medial compartment loading and progressive degeneration [7]. Correction of the coronal axis aims to restore a more physiological distribution of force throughout the limb [11]. Several studies have shown that knee deformity is related to hindfoot position and ankle orientation and that correction after TKA may alter these relationships [12,13,14]. Because the foot is the final point of contact with the ground, plantar load analysis offers a direct measure of how alignment change is expressed distally.

Evidence on plantar loading after TKA is limited and inconsistent, and the early relationship between radiographic correction and functional adaptation of the foot has not been clarified [15,16]. Critically, previous studies have generally compared group means before and after surgery. Such a comparison cannot establish whether patients who receive a larger correction experience a larger change in foot loading, which is the question that a claim of causal linkage requires.

We therefore performed a prospective study with two aims: to determine whether static plantar load distribution changes within the first postoperative month and to test at the level of the individual patient whether the magnitude of coronal correction predicts the magnitude of load change. We hypothesized that restoration of alignment would be accompanied by an early redistribution of plantar load proportional to the correction achieved.

## 2. Materials and Methods

### 2.1. Study Design and Participants

This prospective observational study was conducted in the Department of Orthopaedics and Traumatology, Afyonkarahisar Health Sciences University Faculty of Medicine, between 1 November 2024 and 1 August 2025. Ethical approval was obtained from the institutional Non-Interventional Clinical Research Ethics Committee before commencement (Decision No.: 2024/9, dated 1 November 2024), and all enrolment took place after this date. Consecutive patients undergoing unilateral TKA for advanced primary or secondary knee osteoarthritis were assessed for eligibility. The study is reported in accordance with the STROBE statement [17].

Patients were eligible if they were between 50 and 80 years of age, required unilateral TKA for advanced knee osteoarthritis, were able to stand independently during plantar load assessment, and consented to both preoperative and postoperative pedobarographic measurement. Patients were excluded if they had previous arthroplasty or previous intra-articular surgery of the index knee (a healed extra-articular high tibial osteotomy without retained hardware affecting alignment measurement was not an exclusion), bilateral simultaneous TKA, previous fracture or severe deformity of the ipsilateral lower extremity, advanced ankle or foot deformity capable of independently affecting plantar loading, active inflammatory arthritis or inflammatory involvement of the foot or ankle, peripheral neuropathy or uncontrolled diabetes mellitus with suspected sensory impairment, or any neurological, neuromuscular, vestibular or cognitive condition that impaired the ability to stand unsupported and to load both limbs on instruction at the time of measurement. Stable comorbidities recorded in the medical history that did not meet these operational criteria (for example, quiescent rheumatoid arthritis without foot involvement, episodic vertigo asymptomatic at the time of testing, upper limb essential tremor, or mild cognitive impairment with preserved ability to follow instructions) were not grounds for exclusion. The patients concerned are identified in Section 3.1, and a sensitivity analysis excluding them is reported in Section 3.4.

Sixty-four consecutive patients were enrolled and underwent preoperative assessment. Four patients did not attend the first postoperative month evaluation and were lost to follow-up; all four were women. Sixty patients completed both assessments and were included in the analysis (Figure 1).

### 2.2. Surgical Technique and Rehabilitation

All procedures were performed by the same surgical team using a medial parapatellar approach, conventional instrumentation, and a mechanical alignment philosophy with a target of neutral coronal alignment. A single implant system was used in every patient (ADREMED WIND^®^ total knee prosthesis, ADREMED, Ankara, Türkiye) that was cemented with a posterior-stabilized design; the patella was resurfaced in all cases. Soft tissue balancing was performed by sequential release as required to achieve symmetrical extension and flexion gaps.

All patients followed an identical rehabilitation protocol. Full weight bearing as tolerated was permitted from the first postoperative day, with a walking frame used for the first three postoperative weeks. Range of motion and quadriceps strengthening exercises were commenced during the inpatient stay and continued thereafter. At the time of pedobarographic assessment, all patients stood unsupported without a walking aid.

### 2.3. Radiographic and Clinical Measurements

Coronal alignment was quantified as the hip–knee–ankle (HKA) angle on standing full-length anteroposterior radiographs of both lower limbs, measured between the line joining the center of the femoral head to the center of the intercondylar eminence and the line joining the center of the intercondylar eminence to the center of the talar dome. Varus deviation was recorded as a positive value, and valgus deviation was recorded as a negative value. Signed values were used throughout the analysis. The Q angle was measured radiographically with the patient standing. All radiographic measurements were performed by a single experienced orthopedic surgeon using calibrated digital measurement software. Representative preoperative and postoperative measurements are shown in Figure 2.

Passive knee extension was assessed by the same observer at both time points with the patient supine, the hip in neutral, and a gentle passive extension force applied at the ankle using a standard goniometer. An extension deficit exceeding 5° was regarded as clinically significant. The preoperative assessment was performed under anesthesia immediately before surgery, whereas the postoperative assessment was performed in the awake patient. The assessment technique is illustrated in Figure 3.

### 2.4. Pedobarographic Assessment

Static plantar load analysis was performed with the Gait Scan AS-120 pedobarographic system (Sintech Sağlık Teknolojileri, Istanbul, Türkiye), following established principles of plantar pressure assessment [18]. The platform measures 120 × 40 cm and contains 6864 matrix sensors, giving a spatial resolution of 8 mm and 12-bit data resolution over a measurement range of 0.5–100 psi. Data were sampled at 100 Hz. Measurements were obtained with the patient standing barefoot on a rigid floor surface in a comfortable upright position, gaze fixed straight ahead, and arms relaxed at the sides. Three consecutive recordings were obtained from each patient, separated by rest intervals to limit fatigue and adaptation effects, and the mean of the three recordings was used for analysis. The measurement setup and a representative device output are shown in Figure 4 and Figure 5. All testing was performed on the same device under identical conditions at both time points, with the same standardized positioning.

Before each recording, patients were instructed to distribute their body weight evenly between the two feet. This instruction was applied uniformly at both assessments, and no recording was obtained in a self-selected stance. The implications of this are addressed in the Limitations. Dynamic gait-based pedobarography was not available at our institution during the study period.

**Figure 4:**
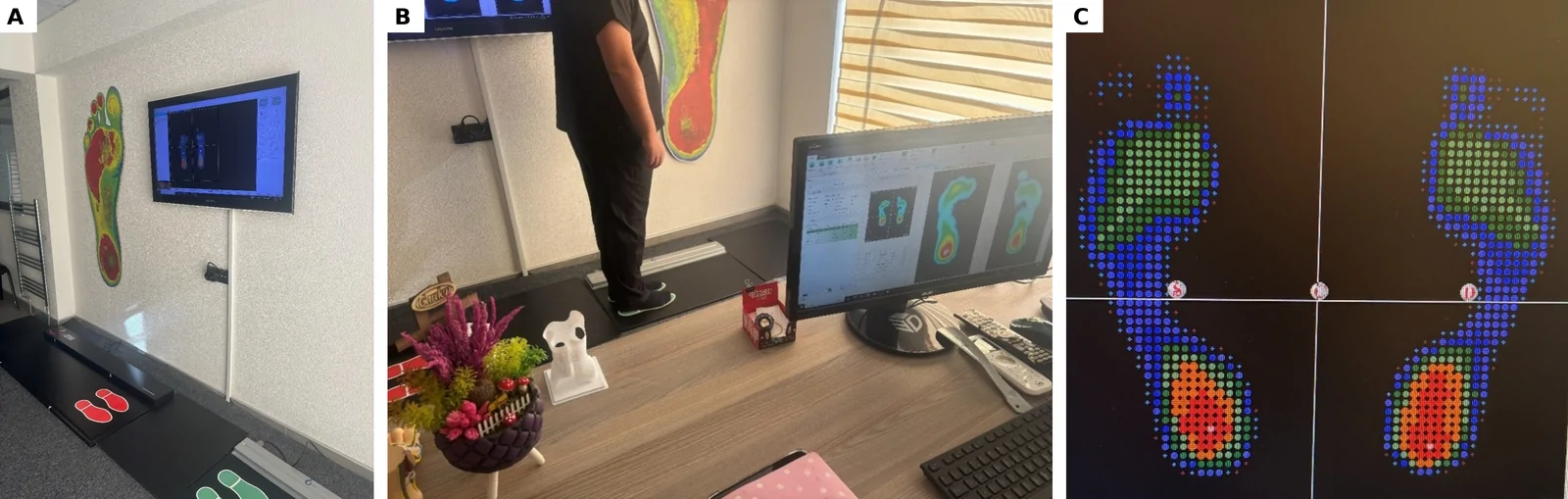
Pedobarographic measurement setup and representative output. (**A**) Gait Scan AS-120 platform with real-time display. (**B**) Standardized patient positioning during static measurement—barefoot, upright, unsupported stance. (**C**) Representative output showing bilateral plantar load distribution with color-coded regions.

**Figure 5:**
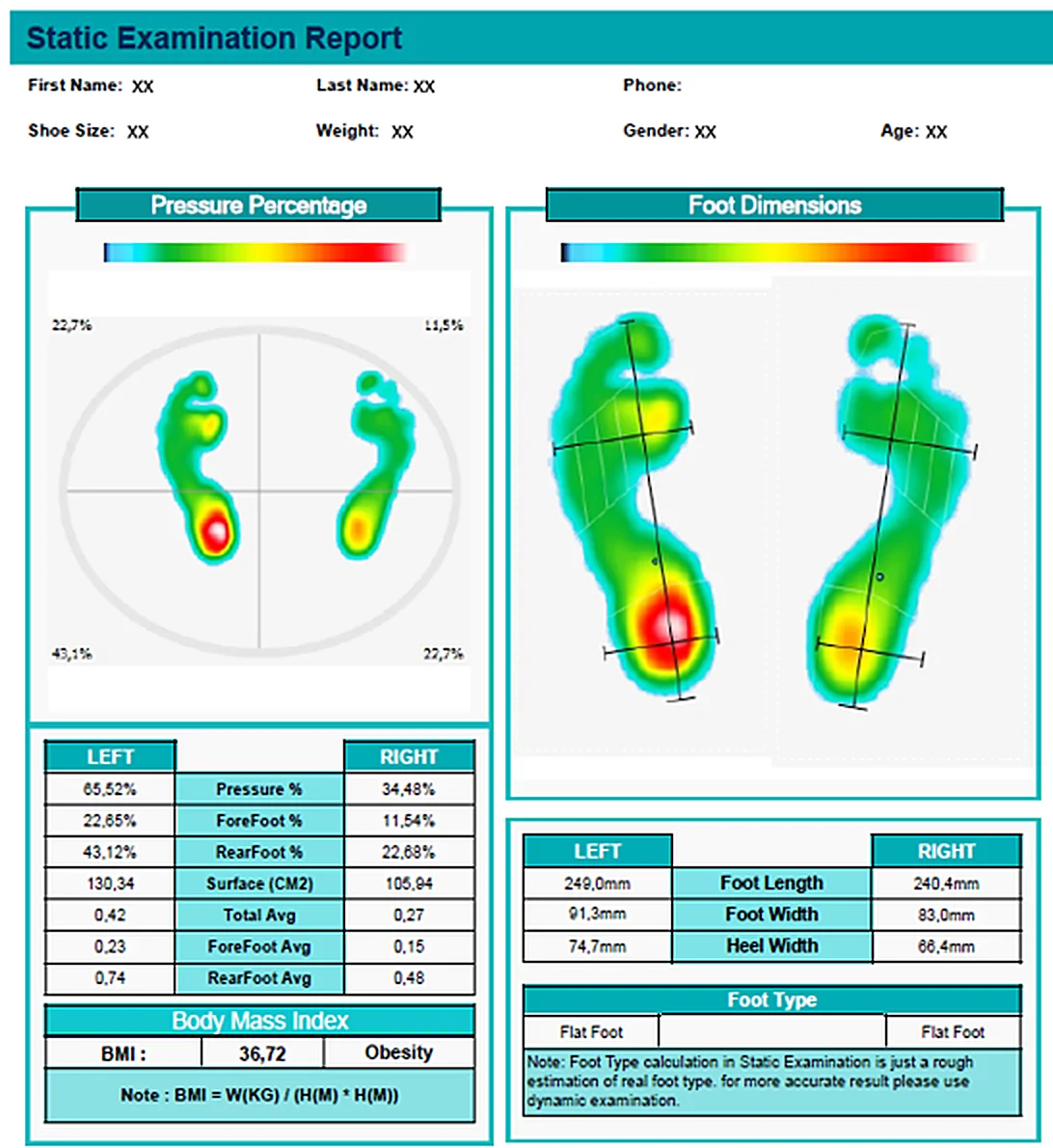
Representative static examination report generated by the Gait Scan AS-120 system, showing the load share percentages and foot dimensions from which the study outcomes were derived. Patient identifiers have been removed. Decimal commas and the asterisk in the body mass index formula appear as generated by the device software and have not been altered.

### 2.5. Outcome Definitions

The primary outcome was the relative bilateral load share of the operative foot defined as the percentage of the total bilateral load borne by that foot, such that the operative and non-operative values sum to 100% by construction. Regional load share was defined analogously for the forefoot and hindfoot as percentages of the same total bilateral load; these regional values are reported by the device software as separate quantities and do not necessarily sum exactly to the total load share of the corresponding foot. All exported values were checked against the original device reports before the revised analysis; transcription errors in the postoperative load share record of three patients were identified and corrected from the source reports. Thus, the operative and non-operative totals sum to 100% in every patient at both time points. These variables express load distribution and are not measures of pressure. As a secondary outcome, regional mean pressure was recorded in kgf/cm^2^ for the total foot, forefoot, and hindfoot of each side. Both sets of variables were derived automatically by the device software from the recorded sensor matrix.

### 2.6. Blinding

Formal blinding of assessors was not implemented. The primary and secondary pedobarographic outcomes were derived automatically using the device software without operator input into the identification of regions or the calculation of values and are therefore observer-independent by construction. Blinding to time point was not feasible for the radiographic and clinical measurements since the prosthesis is visible on postoperative radiographs and an extension deficit is directly apparent on examination. To limit bias, these measurements were performed by a single observer using a standardized protocol. Patient identifiers were removed from exported pedobarographic reports, and the analysis was performed on a coded dataset.

### 2.7. Sample Size and Power

The sample size was determined a priori using G*Power version 3.1.9.7 (Heinrich-Heine-Universität Düsseldorf, Düsseldorf, Germany) [19,20]. The calculation was based on the Wilcoxon signed-rank test (matched pairs), which was selected because normality could not be assumed for all plantar load variables. The calculation implemented in G*Power using the asymptotic relative efficiency (A.R.E.) method with a normal parent distribution, in which the required sample size is derived from the corresponding paired *t* test. The software therefore reports a noncentrality parameter, a critical t value, and non-integer degrees of freedom. Specifying a two-sided α of 0.05, target power of 0.90, and a moderate effect size of d = 0.40—a magnitude regarded as clinically meaningful for functional and biomechanical outcomes in orthopedic research [21]—yielded a noncentrality parameter δ of 2.97, a critical t value of 1.67, 54.39 degrees of freedom, a minimum required sample of 58 patients, and an actual power of 90.2%. Sixty-four patients were enrolled to allow for attrition.

This calculation addressed the detection of a difference and not the demonstration of equivalence. The power of the two one-sided tests procedure for the primary outcome was therefore calculated post hoc, using the observed standard deviation of the within-patient change in load share (12.33 percentage points), *n* = 60, α = 0.05 and a true change of zero. Power was 0.86 for the ±5-percentage point margin (0.64 if the true change equals the observed −1.74 points) and 0.17 for the ±3-point margin, for which approximately 190 patients would have been required. The ±5-point margin is accordingly reported as the primary equivalence analysis and the ±3-point margin as an exploratory sensitivity analysis.

### 2.8. Statistical Analysis

Analyses were performed using IBM SPSS Statistics version 30.0 (IBM Corp., Armonk, NY, USA) and Python 3.12 (SciPy 1.14, statsmodels 0.14). Continuous variables are presented as mean ± standard deviation, and categorical variables are presented as number and percentage. Normality was assessed with the Shapiro–Wilk test, and homogeneity of variance was assessed with Levene’s test.

Within-patient change was assessed with the Wilcoxon signed-rank test, and mean change is reported with a 95% confidence interval and the standardized paired effect size dz. Between-sex comparisons used the Mann–Whitney U test. Because a *p* value above 0.05 does not establish the absence of a meaningful change, two one-sided tests (TOSTs) were additionally applied to the primary and regional load share outcomes using a primary equivalence margin of ±5 percentage points of bilateral load and an exploratory, stricter margin of ±3 percentage points [22].

The ±5-point margin was chosen on three grounds. First, in healthy adults standing quietly, the load borne by each foot is close to 50% but varies between individuals with a standard deviation of approximately 4–5 percentage points, so that a redistribution smaller than 5 points lies within the range of physiological inter-limb asymmetry. In healthy young adults measured on a pressure platform, static load share per foot was 50.2 ± 5.5% (*n* = 77) and 48.6 ± 5.6% in non-athlete controls (*n* = 33), and a per-foot share of 50 ± 5% is used as the reference range for balanced stance [23,24]. Second, a change judged equivalent should not be smaller than the noise of the measurement. Test–retest studies of standing weight-bearing symmetry on dual force platforms report excellent reliability (ICC ≥ 0.89) with a minimal detectable change of approximately 5 percentage points of body weight and of 5.4–7.3 points in patients with impaired postural control [25,26]. The ±5-point margin therefore lies at the limit of what repeated static measurement can distinguish from measurement variability, whereas the ±3-point margin lies within it, which is why three trials were averaged and why the ±3-point margin was treated as exploratory. Third, a change of 5 percentage points corresponds to 0.41 standard deviations of the observed change score, i.e., to the same moderate effect size (d = 0.40) prespecified as the smallest clinically meaningful difference in the sample size calculation. The margin is therefore the equivalence counterpart of the difference the study was designed to detect. Two sensitivity analyses were added at the request of the reviewers. These included exclusion of the seven patients described in Section 2.1 and omission of the postoperative extension deficit from the multivariable model because the preoperative and postoperative extension measurements were obtained under different conditions (Section 2.3).

The relationship between the magnitude of coronal correction and the change in plantar load was examined at the level of the individual patient using Pearson and Spearman correlation of change scores and multivariable linear regression of the change in load share on the magnitude of correction adjusted for age, sex, body mass index, preoperative deformity, and postoperative extension deficit. Variance inflation factors were inspected. Because the two feet and the regional subdivisions are compositional and therefore mathematically dependent, a linear mixed model with a random intercept for patient was fitted with time, side, and sex as fixed effects, and the time × sex interaction was tested explicitly rather than inferred from separate within-sex tests.

Change in the ordinal extension deficit category was analyzed using the McNemar–Bowker test of symmetry and the Stuart–Maxwell test of marginal homogeneity, both of which account for the paired structure of the data, supplemented by a transition table and by the Wilcoxon signed-rank test on patient-level values in degrees. A two-sided *p* value below 0.05 was considered statistically significant.

## 3. Results

### 3.1. Participants

Of 64 consecutive patients enrolled, 60 (93.8%) completed both assessments and were analyzed; four women were lost to follow-up before the first postoperative month (Figure 1). The analyzed cohort comprised 43 women (71.7%) and 17 men (28.3%) with a mean age of 65.4 ± 5.7 years. Fifty-nine patients had varus and one had valgus deformity. In total, 59 were Kellgren–Lawrence grade 4, and one was grade 3. Of note, 58 underwent primary TKA, and two had a previous high tibial osteotomy. The left knee was operated in 34 patients, and the right was operated in 26. Nineteen patients (31.7%) had a previous contralateral TKA. Seven patients had a history that warrants explicit mention in relation to the exclusion criteria: two had undergone a healed high tibial osteotomy of the index knee, one had seropositive rheumatoid arthritis in clinical remission without foot or ankle involvement, two reported episodic vertigo that was asymptomatic at both assessments, one had an upper limb essential tremor, and one had mild cognitive impairment attributed to Alzheimer disease but was able to follow the measurement instructions and to stand unsupported. None met the operational exclusion criteria; all seven are included in the primary analysis and excluded in a sensitivity analysis (Section 3.4). Postoperative assessment was performed at a mean of 30.9 ± 3.3 days (range 25–39 days). Baseline characteristics did not differ significantly between men and women (Table 1). The preoperative Q angle was larger in men, but the difference did not reach statistical significance (*p* = 0.060).

At least one systemic comorbidity was present in 48 patients (80.0%), and more than one was noted in 24 (40.0%). This was most frequently hypertension (55.0%) and diabetes mellitus (33.3%). The full distribution of comorbidities is given in Table 2.

### 3.2. Coronal Alignment and Q Angle

The HKA angle improved from 10.79 ± 4.24° of varus preoperatively to 3.31 ± 3.51° of varus at one month, with a mean correction of 7.48 ± 4.34° (95% CI 6.36 to 8.60; dz = −1.72; *p* < 0.001). Correction was significant in both sexes (men 8.74 ± 5.17°, women 6.98 ± 3.92°; both *p* < 0.001) and in the varus subgroup analyzed separately (10.90 ± 4.20° to 3.40 ± 3.47°; *p* < 0.001). Notwithstanding this improvement, alignment was not restored to neutral in the cohort as a whole. Residual varus was present in 47 of 60 patients (78.3%), whereas 13 were in valgus. Only 26 (43.3%) lay within 3° of neutral (Table 3).

The Q angle increased from 2.64 ± 3.63° to 6.56 ± 2.13° (mean change +3.92°, 95% CI 2.96 to 4.89; dz = +1.05; *p* < 0.001), with significant increases in men (4.44 ± 1.92° to 6.99 ± 2.10°; *p* = 0.004) and women (1.93 ± 3.91° to 6.40 ± 2.14°; *p* < 0.001). The magnitude of coronal correction correlated weakly but significantly with the change in Q angle (r = 0.307, *p* = 0.017).

### 3.3. Extension Deficit

The measured passive extension deficit was 0.33 ± 1.81° preoperatively under anesthesia and 8.17 ± 7.30° at one month in the awake patient, representing an apparent increase of 7.83° (95% CI 5.88 to 9.79; dz = +1.04). Because the preoperative value was obtained under anesthesia and the postoperative value in the awake patient, the magnitude of this change is not a quantitative measure of postoperative deterioration. The former reflects fixed deformity alone, whereas the latter includes pain-related guarding, effusion, and arthrogenic muscle inhibition. The postoperative value is used in the Discussion only as a description of the state of the knee at the time of pedobarography. The paired ordinal analysis confirmed a significant shift in distribution (McNemar–Bowker χ^2^ = 30.35, df = 10, *p* < 0.001; Stuart–Maxwell χ^2^ = 30.20, df = 4, *p* < 0.001; Wilcoxon *p* < 0.001). The transition table shows that 37 of the 58 patients with full preoperative extension developed a deficit of 5° or more, while one of the two patients with a preoperative 10° deficit regained full extension (Table 4). At the time of pedobarographic assessment, 38 patients (63.3%) had a residual deficit. Because the two measurements were obtained under different conditions, this difference describes the state of the knee at the time of pedobarography rather than a quantified deterioration (Section 4.4).

### 3.4. Plantar Load Distribution

Load share of the operative foot did not change significantly between the two assessments (45.42 ± 10.33% to 43.68 ± 9.79%; mean change −1.74 percentage points, 95% CI −4.92 to +1.45; dz = −0.14; *p* = 0.247). Forefoot and hindfoot load share were likewise unchanged, as were all three load share variables of the non-operative foot (Table 5). Regional mean pressure showed the same pattern, with no significant change in any region of either foot (Table 6).

Because these are non-significant results, equivalence was tested formally. Within a margin of ±5 percentage points of bilateral load, the change in total, forefoot, and hindfoot load share of the operative foot was statistically equivalent to zero (TOST *p* = 0.022, *p* < 0.001 and *p* < 0.001 respectively). In the exploratory analysis with the stricter margin of ±3 percentage points, for which the study was underpowered (Section 2.7), equivalence was demonstrated for forefoot (*p* = 0.015) and hindfoot load share (*p* = 0.017) but not for total load share (*p* = 0.216) (Table 7). The latter result is uninformative in either direction.

In the sensitivity analysis excluding the seven patients described in Section 3.1 (*n* = 53), load share of the operative foot changed by −2.71 percentage points (95% CI −6.15 to +0.73; Wilcoxon *p* = 0.097). The 90% confidence interval of this change (−5.59 to +0.17) crossed the lower equivalence bound. Thus, equivalence within ±5 points was not demonstrated in this subgroup (TOST *p* = 0.094), and the correlation between correction magnitude and change in load share remained non-significant (r = 0.248, *p* = 0.074). The direction of all estimates was unchanged, but the equivalence finding is therefore less robust than the full-cohort analysis alone would suggest. This is reflected in the Discussion and Conclusions.

In an exploratory post hoc analysis by postoperative alignment (Supplementary Table S1), load share of the operative foot changed by −0.37 percentage points (95% CI −5.03 to +4.30; Wilcoxon *p* = 0.527) in the 26 patients whose postoperative HKA angle lay within 3° of neutral and by −2.79 points (95% CI −7.31 to +1.74; *p* = 0.256) in the 34 patients with residual malalignment greater than 3°. The two changes did not differ (Mann–Whitney *p* = 0.660). Equivalence within ±5 points was demonstrated in the neutrally aligned subgroup (90% CI −4.24 to +3.51; TOST *p* = 0.026) but not in the malaligned subgroup (90% CI −6.55 to +0.98; *p* = 0.164), and the absolute residual postoperative HKA angle was not correlated with the change in load share (r = −0.069, *p* = 0.601; ρ = 0.000, *p* = 0.998). This analysis was not prespecified and the subgroups are small.

**Table 7:** Equivalence testing (TOST) of the change in relative bilateral load share of the operative foot.

Outcome	Mean Change (%)	90% CI	Margin ± 5%	Margin ± 3% (Exploratory)
Total load share	−1.74	−4.40, +0.92	*p* = 0.022 (equivalent)	*p* = 0.216 (not equivalent)
Forefoot load share	−0.87	−2.46, +0.73	*p* < 0.001 (equivalent)	*p* = 0.015 (equivalent)
Hindfoot load share	−0.69	−2.48, +1.09	*p* < 0.001 (equivalent)	*p* = 0.017 (equivalent)

Two one-sided tests were applied with equivalence margins of ±5 and ±3 percentage points of total bilateral load. Equivalence is declared when the larger of the two one-sided *p* values is below 0.05, which corresponds to the 90% confidence interval lying entirely within the margin. CI: confidence interval.

### 3.5. Relationship Between Correction and Load Change

No statistically significant relationship was demonstrated between the magnitude of coronal correction and the change in load share of the operative foot (r = 0.222, *p* = 0.088; ρ = 0.191, *p* = 0.144), the change in forefoot (r = 0.188, *p* = 0.151) or hindfoot load share (r = 0.172, *p* = 0.190), or the change in total mean pressure (r = 0.009, *p* = 0.948) (Figure 6; Table 8).

In a multivariable model adjusted for age, sex, body mass index, preoperative deformity, and postoperative extension deficit, no significant association between the magnitude of correction and the change in load share was found (β = 0.428 percentage points per degree, 95% CI −0.598 to 1.455; *p* = 0.407). The confidence interval is wide. A 10° correction is compatible with anything between a 6.0-point decrease and a 14.6-point increase in operative-foot load share, so the estimate excludes neither a clinically relevant positive nor a negative association. Omitting the postoperative extension deficit from the model did not materially change the estimate (β = 0.445, 95% CI −0.586 to 1.476; *p* = 0.391). No covariate reached significance, and the model explained little variance (R^2^ = 0.109; adjusted R^2^ = 0.008; F test *p* = 0.388). All variance inflation factors were below 2 (Table 9).

In the linear mixed model of load share with a random intercept for patient, neither the time × sex interaction (β = 0.57, *p* = 0.873) nor the time effect (β = −1.90, *p* = 0.317) was significant, indicating that the absence of change did not differ between men and women. When both feet were entered simultaneously the between-patient variance component approached zero, representing a direct consequence of the compositional constraint that the two load share values sum to 100%; the operative-side model is therefore reported.

## 4. Discussion

The principal finding of this study is that one month after TKA, static plantar load distribution was statistically equivalent to its preoperative state and that no statistically significant relationship was demonstrated between the magnitude of coronal correction achieved in an individual patient and the magnitude of load change. Because the measurement protocol required patients to load both limbs symmetrically, this equivalence is conditional on the protocol. An instruction that constrains asymmetry cannot detect a redistribution that would be expressed only in self-selected stance, and the result may therefore overstate the stability of spontaneous load sharing. The second observation is the more informative of the two, although its precision is limited. A simple before-and-after comparison can show only that group means are similar. By analyzing change scores at the level of the individual patient and by adjusting for age, sex, body mass index, preoperative deformity, and postoperative extension deficit, we were able to test the specific proposition contained in our hypothesis. No statistically significant relationship was demonstrated in the available sample. Patients who received a correction of 15° showed no detectably greater redistribution of foot loading at one month than those corrected by 5°, although the width of the confidence interval means that a proportional effect of modest size cannot be excluded. The unadjusted correlation approached significance (r = 0.222, *p* = 0.088).

We regard the equivalence analysis as an important element of this result. A *p* value above 0.05 indicates only that a difference was not detected, which is compatible both with a true absence of change and with insufficient sensitivity. Formal equivalence testing within a margin of ±5 percentage points of bilateral load allows the stronger and more useful statement that any change occurring in this interval was smaller than a margin we would regard as clinically meaningful. For regional load share, this held within the exploratory margin of ±3 percentage points. The conclusion is necessarily conditional on the margin. At the ±3-point margin, for which the study was underpowered, equivalence of total load share could not be demonstrated. Thus, a redistribution of between 3 and 5 percentage points is neither demonstrated nor excluded by these data, and the term “equivalent” applies at the ±5-point margin only. The grounds for regarding ±5 points as the smallest meaningful redistribution are set out in Section 2.8. Moreover, when seven patients with comorbidities or previous osteotomy were excluded, the 90% confidence interval of the change crossed the ±5-point bound, and equivalence was no longer demonstrated. The finding should therefore be regarded as established for the cohort as a whole but not as robust to reasonable changes in the analyzed population.

### 4.1. Why an Alignment-Driven Change May Not Be Expressed at One Month

Four features of this cohort and protocol each provide a reason why a genuine effect of realignment might not appear at this stage, and we consider that they should be read together rather than as competing explanations.

First, correction was incomplete. Although the mean improvement of 7.48° was substantial and highly significant, the cohort retained a mean residual varus of 3.31°. In total, 78.3% of patients remained in varus, and fewer than half lay within 3° of neutral. The distal consequences of realignment can only be as large as the realignment achieved, and a limb that remains in varus continues to present a broadly similar coronal load line to the foot.

Second, the extension deficit present at the time of assessment. At the one-month visit, 63.3% of patients had a measured deficit of 5° or more. As the preoperative value was obtained under anesthesia, the change from baseline cannot be quantified (Section 3.3), and only the postoperative state is considered here. A knee held in even a few degrees of flexion displaces the ground reaction force vector anteriorly and alters heel-to-forefoot load transfer, and Saito et al. have shown that restriction of knee range of motion shifts the center of plantar pressure in medial knee osteoarthritis [27]. Loss of terminal extension in the early postoperative weeks is a recognized and largely transient phenomenon attributable to periarticular edema, effusion, hamstring spasm, arthrogenic quadriceps inhibition, and pain-related guarding rather than to fixed deformity, and it commonly resolves between the third and sixth postoperative months. The values reported here therefore describe the earliest phase of recovery.

Third, the measurement was static. Static pedobarography reflects postural load sharing at a single instant and is insensitive to the temporal characteristics of loading. Paterson et al. found that static foot posture was unrelated to knee pain in medial knee osteoarthritis, whereas dynamic in-shoe plantar forces correlated with symptom severity [28]. This illustrates the limited explanatory reach of static measurement in this population. Realignment plausibly alters how load is transferred through the foot over the stance phase rather than the proportion borne in quiet standing.

Fourth, and specific to our protocol, patients were instructed before each recording to distribute their weight evenly between the two feet. This instruction was applied identically at both assessments and therefore does not bias the comparison of the two time points. However, it constrains the range over which asymmetry can be expressed, pulls both measurements toward a 50/50 distribution, and thereby increases the probability of a positive equivalence result. It may attenuate precisely the inter-limb difference the study set out to detect. The equivalence demonstrated here is therefore equivalence of instructed symmetrical stance, not of spontaneous load sharing. We regard this as a genuine limitation of the measurement protocol rather than a feature of the biology, and it is discussed further below.

### 4.2. Relation to Previous Work

Restoration of alignment is a principal objective of TKA, and a neutral coronal axis is considered important for load transmission and implant biomechanics [29]. In knee osteoarthritis, varus malalignment increases medial compartment loading and contributes to progressive degeneration [7,30]. Our alignment findings are consistent with this literature in direction, while illustrating that a mechanically aligned technique with conventional instrumentation does not reliably deliver neutrality in every patient.

This has a direct bearing on the generalizability of our findings. Correction was achieved with conventional instrumentation, and only 43.3% of patients were within 3° of neutral postoperatively, a proportion lower than that expected with navigated, robotic-assisted, or patient-specific instrumentation. Because incomplete correction is one of the reasons we advance for the absence of early redistribution (Section 4.1), a cohort in which neutral alignment is reliably restored could in principle show a redistribution that we did not detect. Our exploratory subgroup analysis does not support this however. In the 26 patients whose postoperative axis lay within 3° of neutral, load share of the operative foot was equivalent to baseline within ±5 points and did not differ from the malaligned subgroup (Section 3.4). This argues against incomplete correction as the sole explanation. However, the subgroups are small, and the analysis was not prespecified. Conversely, kinematic alignment pursues a different target, and different plantar loading patterns have been reported after kinematically and mechanically aligned TKA [16,31]. Our results therefore describe a conventionally instrumented, mechanically aligned cohort with frequent residual varus and should not be read as evidence that alignment correction has no distal effect when it is achieved.

Reports of plantar loading after TKA are inconsistent. Some authors have described postoperative alterations in loading patterns attributed to restoration of the mechanical axis [15,32], whereas others have found limited early change [16,31]. Pedobarography has also been applied to the assessment of movement patterns in unstable TKA [33]. Our data are compatible with the latter group and extend their findings by showing that the absence of change is not merely a group-level phenomenon but holds at the level of the individual patient and by quantifying this finding within an equivalence framework rather than reporting a non-significant *p* value alone.

Patients with unilateral knee osteoarthritis commonly develop compensatory loading of the contralateral limb and secondary gait adaptations [34,35,36], and we therefore examined both feet. No change was detected on either side. The compositional structure of these measurements deserves emphasis. The two feet sum to 100% of bilateral load by construction, so a change on one side is necessarily the mirror image of the other, and separate tests of each side convey no independent information. This is why we modelled the two sides jointly and why the between-patient variance component collapsed when we did so.

### 4.3. Confounding, Measurement Error, and the Interpretation of a Non-Significant Result

Confounding and measurement error can bias an estimated association in either direction. A variable associated with both exposure and outcome may inflate an association or, if its effect opposes that of the exposure, attenuate or cancel it. Non-differential measurement error in the exposure or the outcome biases estimates towards the null, and heterogeneity in postoperative pain, strength, and function adds variance that reduces precision. The absence of a statistically significant association therefore cannot be taken as evidence that confounding was absent. In this cohort, the factors most likely to be relevant—postoperative pain, quadriceps weakness, apprehension about loading, and incomplete rehabilitation—would each be expected to act against the expression of alignment-driven redistribution, that is, to mask rather than to create an effect. We regard them as part of the explanation for our finding, while acknowledging that they, together with measurement error in a single static assessment, could equally have concealed a genuine relationship between correction and load redistribution.

Several sources of between-patient variation were held constant by design: a single implant system, a single surgical team, a single alignment philosophy, and an identical rehabilitation protocol were used throughout. Others were examined directly. Body mass index did not change over the study period and did not differ between sexes. Comorbidity burden was unrelated to any outcome. The interval between surgery and assessment did not correlate with alignment or load values. Preoperative deformity severity, age, sex, and the postoperative extension deficit were entered into the multivariable model, in which none reached significance.

What these analyses cannot exclude is that a genuine but small redistribution occurred and was obscured by the combined influence of incomplete correction, extension deficit, static measurement, and the weight-sharing instruction. This possibility is why our conclusion is framed as equivalence within a defined margin at one month rather than as evidence that alignment correction has no distal effect.

### 4.4. Strengths and Limitations

The strengths of this study include its prospective consecutive design, the a priori sample size calculation, the uniformity of implant, surgical team and rehabilitation protocol, the assessment of both limbs, the analysis of individual change scores rather than group means alone, and the use of equivalence testing and methods appropriate to paired and compositional data. Several limitations must nonetheless be acknowledged.

First, assessment was confined to a single early time point at a mean of 30.9 days. The design can establish that the measured variables were equivalent at one month. However, it cannot characterize the time course of adaptation, and it does not permit any statement about longer-term remodeling. A design incorporating six weeks, three months, six months, and one year would be required for that purpose.

Second, plantar load was assessed only in static bilateral stance, and patients were instructed to share weight evenly. These findings therefore cannot be extended to dynamic plantar pressure, gait symmetry, center-of-pressure progression, walking kinetics, or neuromuscular adaptation, all of which may change while static load sharing does not. We regard the combination of static measurement and a standardized weight-sharing instruction as the principal methodological limitation of this study. Self-selected stance was not recorded at either assessment, so the extent to which the instruction attenuated asymmetry cannot be estimated from these data; a paired recording in self-selected stance should be regarded as the minimum requirement for any future study of spontaneous load sharing.

Third, postoperative pain scores, analgesic use, quadriceps strength, disease-specific functional scores, walking aid use beyond the protocol, rehabilitation adherence, and weight-bearing tolerance were not part of the study protocol and were not recorded. Foot posture, hindfoot alignment, ankle symptoms, and the radiographic status of the contralateral knee were likewise not assessed. Residual confounding by these variables cannot be excluded, and their measurement is the most important addition required in future work.

Fourth, the a priori sample size calculation addressed the detection of a moderate difference (dz = 0.40) and not equivalence. A post hoc calculation using the observed variability showed adequate power (0.86) for the ±5-point equivalence margin but not for the ±3-point margin (0.17), which is why the latter is reported as exploratory. Sex-specific analyses were not prespecified as primary comparisons and are exploratory. The male subgroup of 17 patients in particular has limited sensitivity, which is one reason the formal time × sex interaction test is reported in preference to separate within-sex comparisons.

Fifth, formal blinding of assessors was not implemented and was not feasible for the radiographic and clinical measurements, although the pedobarographic outcomes are device derived and observer independent. Intra-observer and inter-observer reliability of the radiographic measurements were not assessed, and this should be included in future studies.

Sixth, the preoperative extension assessment was performed under anesthesia whereas the postoperative assessment was performed in the awake patient. Because anesthesia abolishes protective muscle guarding, preoperative deficit may have been underestimated and the magnitude of the apparent increase correspondingly overstated. For this reason, the change in extension deficit is not used as a quantified outcome. The postoperative value, measured at the same visit as the pedobarography, is used only to describe the state of the knee at the time of measurement and as a covariate, and the model was also run without it (Section 3.5). The change in extension deficit reported in Section 3.3 should therefore be regarded as an artifact of measurement conditions as much as of surgery and is not used as evidence of postoperative deterioration.

Seventh, alignment restoration was incomplete. In total, 78.3% of patients retained residual varus, and fewer than half lay within 3° of neutral. Although the exploratory subgroup within 3° of neutral showed the same absence of early redistribution, the findings cannot be extrapolated with confidence to modern TKA cohorts with more accurate restoration of the mechanical axis or to kinematically aligned TKA.

Finally, this was a single-center study of moderate size with a cohort that was almost exclusively varus and Kellgren–Lawrence grade 4, which limits generalizability to valgus deformity and to less advanced disease.

## 5. Conclusions

One month after total knee arthroplasty for advanced, predominantly varus knee osteoarthritis, static bilateral plantar load sharing, measured under an instruction to load both limbs symmetrically, was statistically equivalent to its preoperative state within a margin of ±5 percentage points of bilateral load in the full cohort. This equivalence of instructed symmetrical stance may in part reflect the standardized instruction rather than the absence of a biological effect. However, this equivalence was not maintained in a sensitivity analysis excluding seven patients with comorbidities, and no statistically significant relationship between the magnitude of coronal correction achieved in an individual patient and the magnitude of load change was demonstrated. However, the precision of this estimate was limited. These findings are confined to the first postoperative month, to instructed symmetrical static stance, and to a mechanically aligned technique in which correction was often incomplete. They cannot be extrapolated to cohorts with more accurate alignment restoration, and they do not extend to dynamic plantar loading, gait, or center-of-pressure behavior. They are consistent with the incomplete correction achieved in this cohort, in which mean residual varus was 3.31°, and with the transient extension deficit present in 63.3% of patients at the time of assessment. Whether coronal realignment produces a measurable redistribution of plantar load at a later stage, or under dynamic conditions, remains to be determined by studies incorporating serial follow-up, gait-based pedobarography, and concurrent measurement of pain, muscle strength, and function.

## Figures and Tables

**Figure 1:**
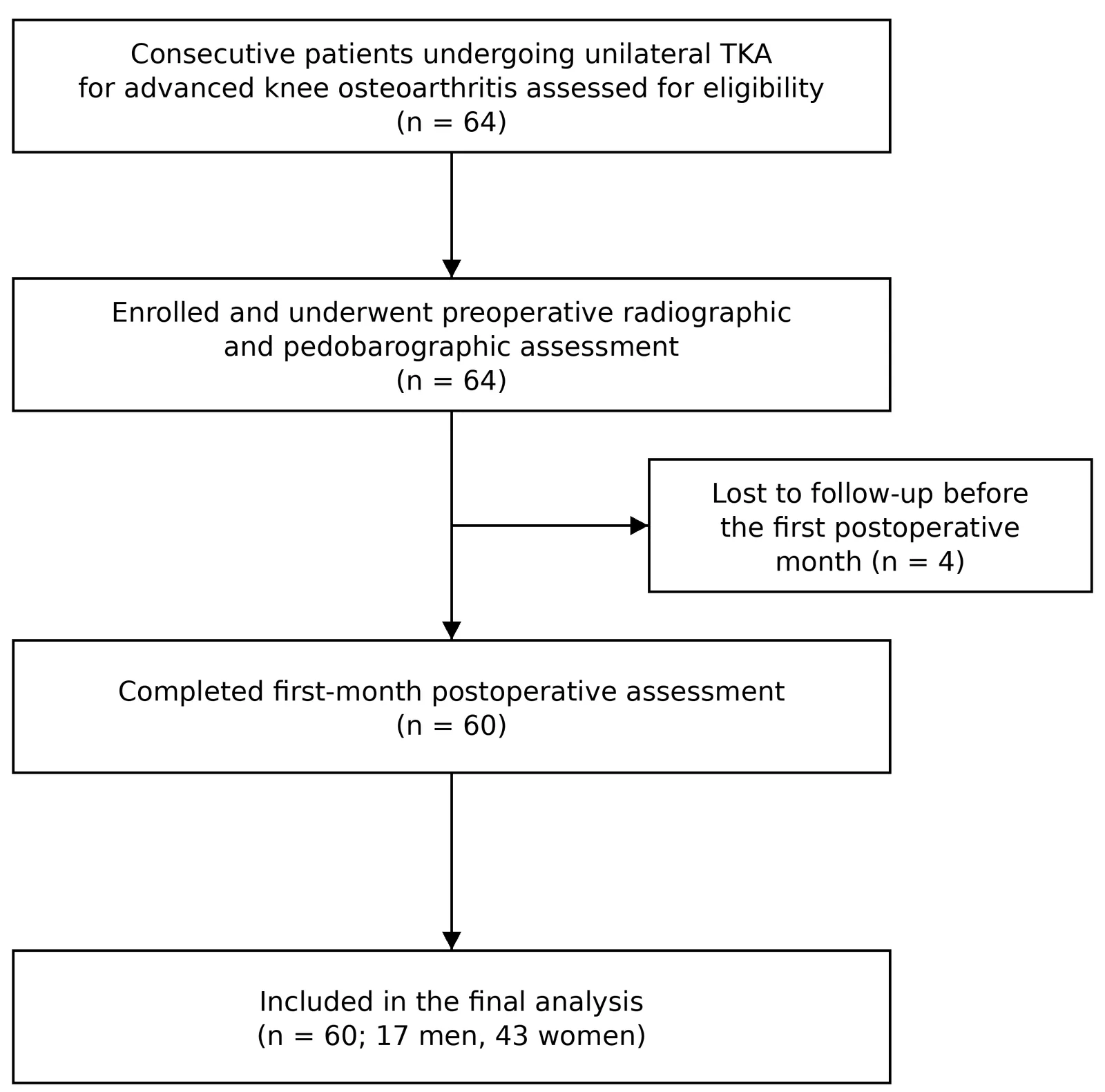
Flow of participants through the study reported in accordance with the STROBE statement.

**Figure 2:**
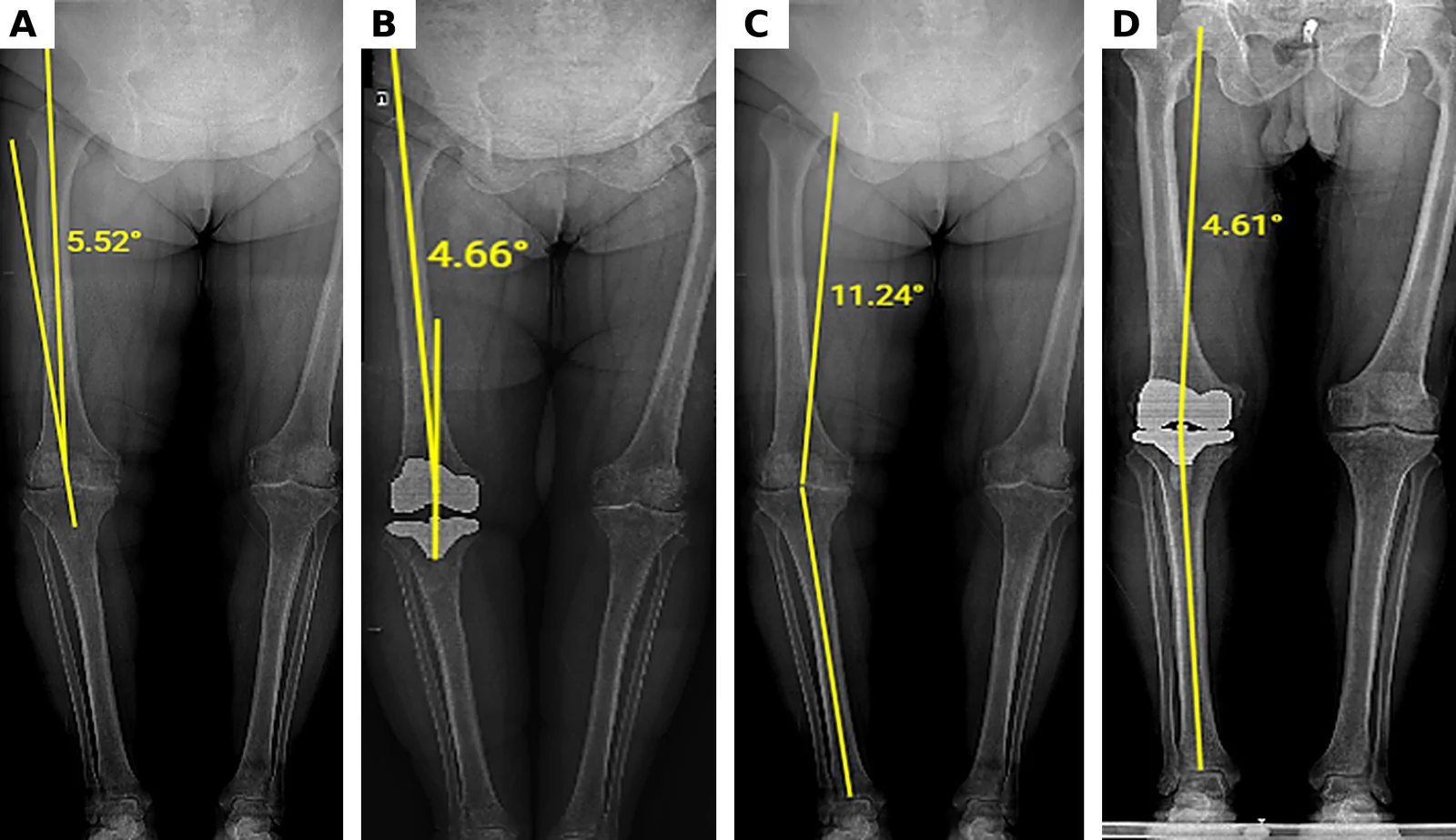
Representative full-length standing radiographs. (**A**) Preoperative Q angle measurement of 5.52°. (**B**) Postoperative Q angle measurement of 4.66°. (**C**) Preoperative hip–knee–ankle angle of 11.24° varus. (**D**) Postoperative hip–knee–ankle angle of 4.61° varus. Yellow lines indicate the measured axes. These images illustrate individual cases and do not represent cohort means.

**Figure 3:**
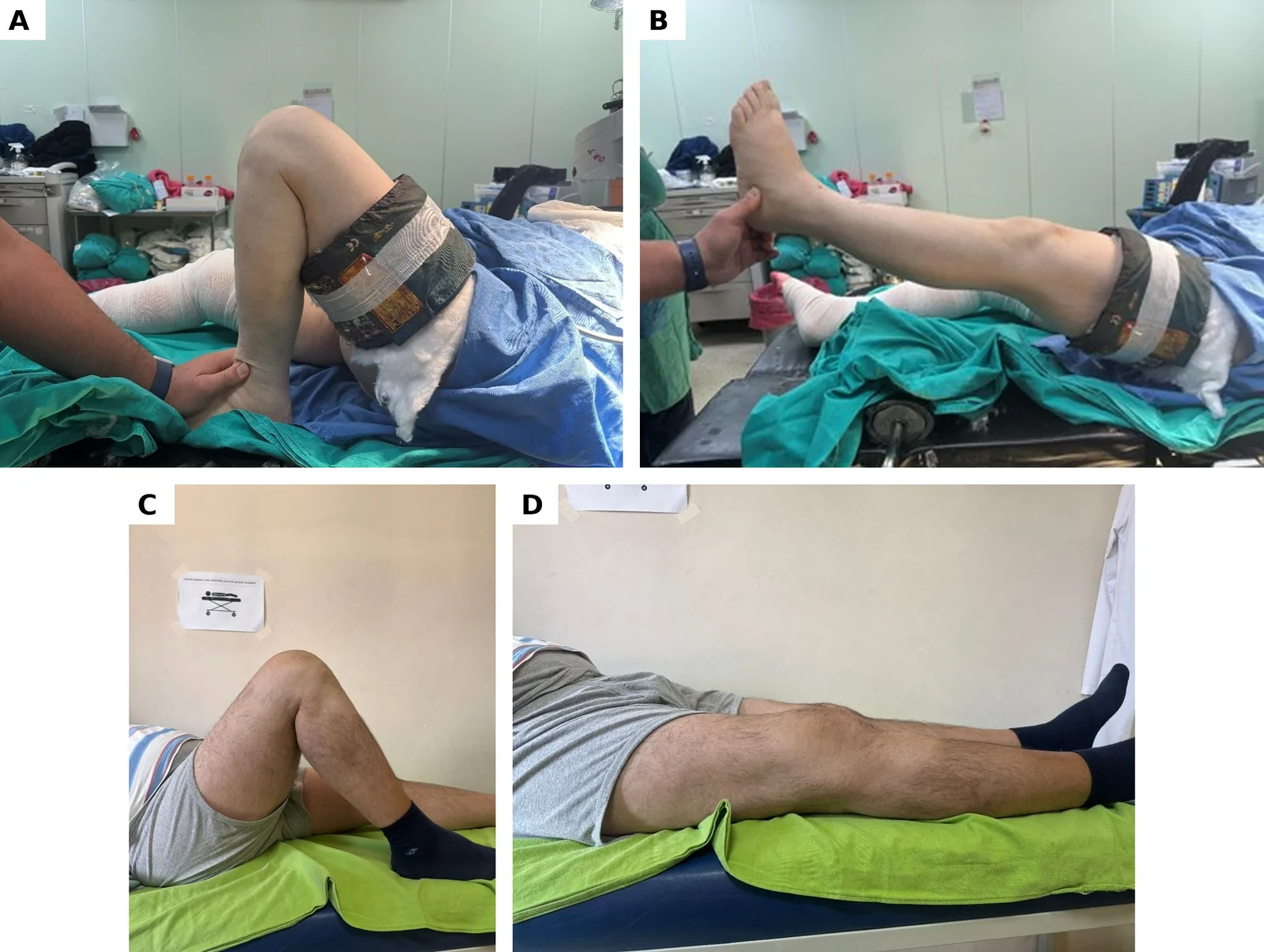
Clinical assessment of passive knee extension. (**A**,**B**) Early postoperative inpatient assessment. (**C**,**D**) First postoperative month outpatient assessment. These images illustrate the goniometric method used to determine the presence and degree of extension deficit.

**Figure 6:**
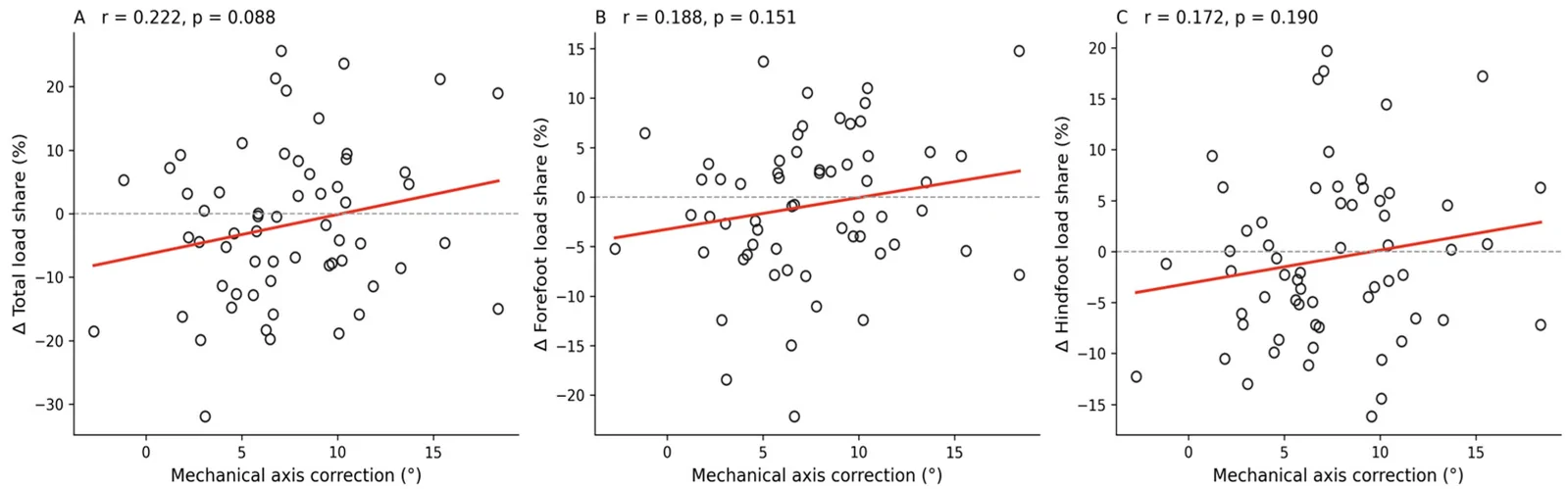
Relationship between the magnitude of coronal correction and the change in relative bilateral load share of the operative foot. (**A**) Total load share. (**B**) Forefoot load share. (**C**) Hindfoot load share. Each point represents one patient; the solid line is the least-squares regression line, and the dashed line indicates zero change. Correlations were non-significant for all three outcomes.

**Table 1:** Baseline characteristics of the analyzed cohort, overall and by sex.

Characteristic	All (*n* = 60)	Men (*n* = 17)	Women (*n* = 43)	*p*
Age (years)	65.40 ± 5.66	66.94 ± 4.21	64.79 ± 6.08	0.211
Body mass index (kg/m^2^)	33.16 ± 5.38	33.01 ± 5.71	33.22 ± 5.32	0.806
Operated side, right/left	26/34	10/7	16/27	0.128
Kellgren–Lawrence grade 4, *n* (%)	59 (98.3)	17 (100)	42 (97.7)	1.000
Varus/valgus deformity	59/1	17/0	42/1	1.000
Primary TKA/previous HTO	58/2	16/1	42/1	0.492
Previous contralateral TKA, *n* (%)	19 (31.7)	6 (35.3)	13 (30.2)	0.703
≥1 Systemic comorbidity, *n* (%)	48 (80.0)	—	—	—
Preoperative HKA angle (° varus)	10.79 ± 4.24	11.23 ± 4.48	10.62 ± 4.19	0.825
Preoperative Q angle (°)	2.64 ± 3.63	4.44 ± 1.92	1.93 ± 3.91	0.060
Preoperative extension deficit (°)	0.33 ± 1.81	0.00 ± 0.00	0.47 ± 2.13	0.464
Interval to postoperative assessment (days)	30.90 ± 3.31	30.82 ± 3.30	30.93 ± 3.36	0.779

Values are mean ± standard deviation or number (%). Between-sex comparisons used the Mann–Whitney U test for continuous variables and the chi-square or Fisher exact test for categorical variables. HKA: hip–knee–ankle; TKA: total knee arthroplasty; HTO: high tibial osteotomy. Varus is expressed as a positive value.

**Table 2:** Distribution of systemic comorbidities (*n* = 60).

Comorbidity	*n* (%)	Comorbidity	*n* (%)
Hypertension	33 (55.0)	Chronic obstructive pulmonary disease	2 (3.3)
Diabetes mellitus	20 (33.3)	Hyperlipidemia	1 (1.7)
Asthma	5 (8.3)	Hypothyroidism	1 (1.7)
Hyperthyroidism	4 (6.7)	Rheumatoid arthritis	1 (1.7)
Goiter	4 (6.7)	Chronic kidney disease	1 (1.7)
Congestive heart failure	2 (3.3)	Arrhythmia	1 (1.7)
Vertigo	2 (3.3)	Valvular insufficiency	1 (1.7)
≥1 Comorbidity	48 (80.0)	No comorbidity	12 (20.0)
>1 Comorbidity	24 (40.0)	Other single conditions *	5 (8.3)

* Immune thrombocytopenic purpura, essential tremor, Alzheimer disease, and idiopathic pulmonary fibrosis, each in one patient. The patients with rheumatoid arthritis (in remission, no foot involvement), vertigo, essential tremor, and Alzheimer disease did not meet the operational exclusion criteria (Section 2.1) and are addressed in a sensitivity analysis (Section 3.4). Comorbidity burden was not associated with alignment, extension deficit, or any plantar loading outcome (all *p* > 0.05).

**Table 3:** Coronal alignment and Q angle before and one month after total knee arthroplasty.

Parameter	Group	Preoperative	Postoperative	Mean Change (95% CI)	dz	*p*
HKA angle (°)	All (*n* = 60)	10.79 ± 4.24	3.31 ± 3.51	−7.48 (−8.60, −6.36)	−1.72	<0.001
	Men (*n* = 17)	11.23 ± 4.48	2.49 ± 3.79	−8.74 (−11.40, −6.08)	−1.69	<0.001
	Women (*n* = 43)	10.62 ± 4.19	3.64 ± 3.38	−6.98 (−8.18, −5.77)	−1.78	<0.001
Q angle (°)	All (*n* = 60)	2.64 ± 3.63	6.56 ± 2.13	+3.92 (+2.96, +4.89)	+1.05	<0.001
	Men (*n* = 17)	4.44 ± 1.92	6.99 ± 2.10	+2.55 (+1.09, +4.00)	+0.90	0.004
	Women (*n* = 43)	1.93 ± 3.91	6.40 ± 2.14	+4.47 (+3.25, +5.68)	+1.13	<0.001

Values are mean ± standard deviation. Varus is expressed as a positive value, and valgus as a negative value. Within-group comparisons used the Wilcoxon signed-rank test. Postoperatively, residual varus was present in 47 of 60 patients (78.3%), and valgus was noted in 13 (21.7%). In total, 26 patients (43.3%) were within 3° of neutral. CI: confidence interval; HKA: hip–knee–ankle.

**Table 4:** Transition of passive extension deficit category between the preoperative and one-month assessments.

Preoperative	0°	5°	10°	15°	20°	Total
0°	21	4	16	9	8	58
5°	0	0	0	0	0	0
10°	1	0	0	1	0	2
15°	0	0	0	0	0	0
20°	0	0	0	0	0	0
Total	22	4	16	10	8	60

Rows indicate the preoperative category and columns the postoperative category; cells give the number of patients. McNemar–Bowker test of symmetry χ^2^ = 30.35, df = 10, *p* < 0.001; Stuart–Maxwell test of marginal homogeneity χ^2^ = 30.20, df = 4, *p* < 0.001; Wilcoxon signed-rank test on patient-level values *p* < 0.001. Mean deficit increased from 0.33 ± 1.81° to 8.17 ± 7.30° (mean change +7.83°, 95% CI +5.88 to +9.79). The preoperative assessment was performed under anesthesia and the postoperative assessment in the awake patient.

**Table 5:** Relative bilateral load share before and one month after total knee arthroplasty (*n* = 60).

Foot and Region	Preoperative (%)	Postoperative (%)	Mean Change (95% CI)	dz	*p*
Operative—total	45.42 ± 10.33	43.68 ± 9.79	−1.74 (−4.92, +1.45)	−0.14	0.247
Operative—forefoot	20.57 ± 5.72	19.70 ± 6.09	−0.87 (−2.78, +1.04)	−0.12	0.446
Operative—hindfoot	24.71 ± 7.09	24.02 ± 7.22	−0.69 (−2.83, +1.44)	−0.08	0.296
Non-operative—total	54.58 ± 10.33	56.32 ± 9.79	+1.74 (−1.45, +4.92)	+0.14	0.247
Non-operative—forefoot	23.47 ± 5.69	24.01 ± 5.15	+0.54 (−1.01, +2.08)	+0.09	0.536
Non-operative—hindfoot	31.27 ± 8.31	32.28 ± 8.58	+1.00 (−1.28, +3.28)	+0.11	0.349

Values are mean ± standard deviation and expressed as a percentage of total bilateral load. The total values of the operative and non-operative feet sum to 100% by construction; regional values are reported independently by the device software and do not necessarily sum to the corresponding total. Within-group comparisons used the Wilcoxon signed-rank test. dz: standardized paired effect size; CI: confidence interval.

**Table 6:** Regional mean plantar pressure before and one month after total knee arthroplasty.

Foot and Region	Preoperative	Postoperative	Mean Change (95% CI)	*p*
Operative—total	0.318 ± 0.072	0.301 ± 0.074	−0.017 (−0.037, +0.003)	0.113
Operative—forefoot	0.250 ± 0.065	0.238 ± 0.071	−0.011 (−0.032, +0.009)	0.450
Operative—hindfoot	0.423 ± 0.159	0.402 ± 0.158	−0.021 (−0.067, +0.025)	0.314
Non-operative—total	0.369 ± 0.087	0.361 ± 0.076	−0.008 (−0.032, +0.015)	0.577
Non-operative—forefoot	0.266 ± 0.072	0.263 ± 0.069	−0.002 (−0.022, +0.017)	0.919
Non-operative—hindfoot	0.538 ± 0.219	0.523 ± 0.194	−0.015 (−0.062, +0.032)	0.745

Values are mean ± standard deviation in kgf/cm^2^. Within-group comparisons used the Wilcoxon signed-rank test. The mean pressure values of the three patients whose load share records were corrected were verified against the device reports and were unchanged.

**Table 8:** Patient-level correlation between the magnitude of coronal correction and change in plantar loading.

Relationship	Pearson r	*p*	Spearman ρ	*p*
Correction vs. Δ total load share	+0.222	0.088	+0.191	0.144
Correction vs. Δ forefoot load share	+0.188	0.151	+0.210	0.107
Correction vs. Δ hindfoot load share	+0.172	0.190	+0.169	0.196
Correction vs. Δ total mean pressure	+0.009	0.948	+0.049	0.708
Correction vs. Δ forefoot mean pressure	−0.040	0.761	−0.011	0.931
Correction vs. Δ hindfoot mean pressure	+0.055	0.674	+0.064	0.629
Correction vs. Δ Q angle	+0.307	0.017	+0.259	0.046
Correction vs. Δ extension deficit	−0.138	0.294	−0.131	0.320

Correction is the reduction in hip–knee–ankle angle between the preoperative and postoperative measurements. All values refer to the operative foot. Δ denotes the postoperative value minus the preoperative value.

**Table 9:** Multivariable linear regression of the change in relative bilateral load share of the operative foot.

Predictor	β	95% CI	*p*	VIF
Magnitude of coronal correction (°)	0.428	−0.598, 1.455	0.407	1.93
Age (years)	−0.193	−0.856, 0.470	0.561	1.37
Male sex	−0.487	−8.068, 7.093	0.898	1.15
Body mass index (kg/m^2^)	0.182	−0.426, 0.790	0.551	1.04
Preoperative HKA angle (°)	0.064	−0.953, 1.082	0.900	1.81
Postoperative extension deficit (°)	−0.303	−0.797, 0.192	0.225	1.27

*n* = 60. Model R^2^ = 0.109, adjusted R^2^ = 0.008, F test *p* = 0.388. Omitting the postoperative extension deficit gave β = 0.445 (95% CI −0.586 to 1.476; p = 0.391) for the magnitude of correction. β: unstandardized regression coefficient; CI: confidence interval; VIF: variance inflation factor; HKA: hip–knee–ankle.

## Data Availability

The dataset analyzed during the current study is available from the corresponding author on reasonable request. All data were anonymized before analysis.

## Supplementary Materials

The following supporting information can be downloaded at: https://www.mdpi.com/article/10.3390/medicina62101840/s1, Supplementary Table S1. Exploratory post hoc analysis of the change in relative bilateral load share of the operative foot by postoperative coronal alignment (n = 60).
