# Supplementary material for: Static Bilateral Plantar Load Sharing One Month After Total Knee Arthroplasty for Varus Osteoarthritis: A Prospective Pedobarographic Equivalence Study
Source: Medicina (Kaunas). 2026 Sep 23;62(10):1840. doi: 10.3390/medicina62101840 (PMC13707244; doi:10.3390/medicina62101840)
Supplement: Supplementary file 1 [file medicina-62-01840-s001.zip › medicina-4569724-supplementary.pdf]

Supplementary Table S1

Supplementary Table S1. Exploratory post hoc analysis of the change in relative bilateral load share of the operative foot by postoperative coronal alignment (n = 60).

	Within 3° of neutral (n = 26)	Residual malalignment > 3° (n = 34)	Between-subgroup p
Preoperative HKA angle (°)	9.97 ± 4.15	11.41 ± 4.27	—
Postoperative HKA angle (°)	0.47 ± 1.43	5.49 ± 3.03	—
Coronal correction (°)	9.51 ± 3.82	5.92 ± 4.11	0.001
Operative-foot load share, preoperative (%)	46.85	44.32	—
Operative-foot load share, postoperative (%)	46.49	41.54	—
Mean change (percentage points)	−0.37	−2.79	0.660
95% CI of change	−5.03 to +4.30	−7.31 to +1.74	
Wilcoxon signed-rank p	0.527	0.256	
Effect size dz	−0.03	−0.21	
90% CI of change (TOST)	−4.24 to +3.51	−6.55 to +0.98	
TOST p, margin ±5 points	0.026 (equivalent)	0.164 (not equivalent)	
TOST p, margin ±3 points (exploratory)	0.128 (not equivalent)	0.462 (not equivalent)	

Values are mean ± standard deviation or mean. Varus is positive and valgus negative. Between-subgroup comparisons used the Mann–Whitney U test. Equivalence is declared when the larger of the two one-sided p values is below 0.05 (90% CI within the margin). Correlation of the absolute residual postoperative HKA angle with the change in load share across all 60 patients: Pearson r = −0.069 (p = 0.601), Spearman ρ = 0.000 (p = 0.998). Subgroups were defined post hoc and the analysis was not prespecified. CI: confidence interval; HKA: hip–knee–ankle; TOST: two one-sided tests.
